# In the twilight zone of protein sequence homology: do protein language models learn protein structure?

**DOI:** 10.1093/bioadv/vbae119

**Published:** 2024-08-17

**Authors:** Anowarul Kabir, Asher Moldwin, Yana Bromberg, Amarda Shehu

**Affiliations:** Department of Computer Science, George Mason University, Fairfax, VA 22030, United States; Department of Computer Science, George Mason University, Fairfax, VA 22030, United States; Department of Computer Science, Emory University, Atlanta, GA 30307, United States; Department of Computer Science, George Mason University, Fairfax, VA 22030, United States

## Abstract

**Motivation:**

Protein language models based on the transformer architecture are increasingly improving performance on protein prediction tasks, including secondary structure, subcellular localization, and more. Despite being trained only on protein sequences, protein language models appear to implicitly learn protein structure. This paper investigates whether sequence representations learned by protein language models encode structural information and to what extent.

**Results:**

We address this by evaluating protein language models on remote homology prediction, where identifying remote homologs from sequence information alone requires structural knowledge, especially in the “twilight zone” of very low sequence identity. Through rigorous testing at progressively lower sequence identities, we profile the performance of protein language models ranging from millions to billions of parameters in a zero-shot setting. Our findings indicate that while transformer-based protein language models outperform traditional sequence alignment methods, they still struggle in the twilight zone. This suggests that current protein language models have not sufficiently learned protein structure to address remote homology prediction when sequence signals are weak.

**Availability and implementation:**

We believe this opens the way for further research both on remote homology prediction and on the broader goal of learning sequence- and structure-rich representations of protein molecules. All code, data, and models are made publicly available.

## 1 Introduction

An explosion in the number of known protein sequences is allowing researchers to harness recent breakthroughs in Natural Language Processing (NLP) due to language models (LMs) and propose Protein Language Models (PLMs) (Heinzinger *et al.* 2019, Bepler and Berger 2021, Elnaggar *et al.* 2022). Like their counterparts in NLP, from BERT (Devlin *et al.* 2019) to GPT-4 (OpenAI *et al.* 2024), PLMs are trained in a semi-supervised fashion by randomly masking out amino-acid tokens or spans of tokens within protein sequences extracted from large protein sequence databases (Steinegger *et al.* 2019, The UniProt Consortium 2020). The model’s objective is to predict the missing amino acids based on the context provided by the surrounding unmasked tokens (Vaswani *et al.* 2017). Key to accomplishing this objective is the ability to weigh the importance of different portions of the input sequence. The introduction of the self-attention mechanism in the transformer architecture allows models to learn these weights and effectively capture the contextual information in input data. In this process, referred to as pre-training, the model builds complex, high-dimensional representations of input sequences (and even individual tokens) (Vaswani *et al.* 2017). The representations learned during pre-training are task-agnostic, which, in principle, through fine-tuning, enables their use in a variety of downstream prediction tasks.

Protein sequence representations learned via PLMs have been shown useful for various prediction tasks, including predicting secondary structure (Elnaggar *et al.* 2022), subcellular localization (Stärk *et al.* 2021, Elnaggar *et al.* 2022), evolutionary relationships within protein families (Hie *et al.* 2022), and Superfamily (Kabir and Shehu 2022) and Family (Nambiar *et al.* 2020) membership. In particular, PLMs are reported to implicitly learn structural information even when trained solely on sequence data (Rao *et al.* 2019, Heinzinger *et al.* 2019, Rives *et al.* 2021, Elnaggar *et al.* 2022). For instance, work in Rao *et al.* (2019) shows that sequence-learned representations confer high performance on an array of downstream protein-structure related tasks, including secondary structure prediction, homology detection, and protein engineering. Rives *et al.* (2021) also tout the utility of sequence representations learned from their Evolutionary Scale Modeling-1 (ESM-1) PLMs for predicting secondary structure, homology, long-range residue contacts, and mutational effects. In Lin *et al.* (2022) the authors introduce a Family of ESM2 models ranging in size from 8M to 15B parameters and utilize their representations for tertiary structure prediction through an equivariant neural network. Though the reported accuracy falls short of the state-of-the-art (SOTA) AlphaFold2 (Jumper *et al.* 2021), models beyond 150M parameters are shown to outperform smaller ones.

A growing argument in the scientific community is that PLMs implicitly learn structure due to their ability to ingest millions of protein sequences, something that was not possible before with methods based on sequence alignment. The hypothesis is that this ability in turn enables PLMs to capture the selective pressures exerted on protein sequences throughout billions of years of evolution (Marquet *et al.* 2021). Note, however, that these pressures come directly from function through structure down to sequence. Function evolves slower than structure, and structure evolves slower than sequence (Illergård *et al.* 2009). Structure and function are well preserved above 30% sequence identity (Rost 1999). Proteins with similar structure and function are indeed present below this level of identity (the “twilight zone”) but cannot be detected from sequence similarity alone (Rost 1999). We refer to these proteins as remote homologs (Rost 1999, Strodthoff *et al.* 2020).

Do PLM-learned sequence representations additionally encode protein structure and to what extent? In this paper, we answer this question by stress-testing PLM-learned representations on a hallmark problem in computational biology, remote homology prediction. The core task is to determine from sequence information alone that two given proteins are remote homologs. In the twilight zone of sequence homology, structure information is essential to address this task, which becomes increasingly challenging as sequence identity decreases.

In this paper, we evaluate representative SOTA transformer-based PLMs in the zero-shot setting on the problem of remote homology prediction at increasing levels of difficulty. The zero-shot setting refers to the fact that we do not fine-tune models on a particular task but directly utilize representations learned by a PLM after pre-training. The evaluation is carried out over decreasing levels of sequence identity. In this manner, we systematically remove sequence-based determinants of homology and so are left with increasingly challenging instantiations of remote homology prediction, where structural knowledge is key to performance.

While advancing remote homology prediction is an active area of research with an increasing number of models and methodologies (Hamamsy *et al.* 2023, Kaminski *et al.* 2023, Kilinc *et al.* 2023, Johnson *et al.* 2024, Liu *et al.* 2024) we focus here on the following SOTA PLMs: TAPE-BERT (Rao *et al.* 2019), Protein-BERT (Brandes *et al.* 2022), ESM1b (Rives *et al.* 2021), ESM2 (Lin *et al.* 2022), Prottrans-BERT, Prottrans-Albert, and Prottrans-T5 (Elnaggar *et al.* 2022). TAPE-BERT is among the first pre-trained PLMs (containing 38M parameters) that is rigorously evaluated and shown effective on a variety of protein prediction tasks. Protein-BERT is a smaller model of 16M parameters that utilizes both protein sequence data and Gene Ontology annotations of sequences during its pre-training. ESM1b is reported by the authors to be the most powerful in the ESM-1 suite of models. The ESM2 model (of 650M parameters) we select is a representative of the top three models (of size 650M, 3B, and 15B parameters) in the ESM2 suite (Lin *et al.* 2022). The Prottrans models we select range in size from 224M to 3B parameters and represent transformer-based PLMs shown powerful in a variety of downstream protein prediction tasks.

To provide a baseline for the observed performance, we use HHblits (Remmert *et al.* 2012), a classic, pre-PLM method for remote homology prediction. HHblits relies on sequence alignment within a hidden Markov model framework. Its utilization as a baseline provides us with a better understanding of the performance gains obtained by the shift away from sequence alignment to PLM-learned sequence representations.

A key contribution of this paper is the rigorous evaluation of small-to-large scale SOTA PLMs on the remote homology prediction task over two datasets, the manually curated SCOP2 dataset (Andreeva *et al.* 2013, 2019) and its extension SCOPe dataset (Fox *et al.* 2014, Chandonia *et al.* 2022). These datasets provide structural and functional categorizations that permit rigorous evaluation of PLM-learned representations along a variety of classic machine learning metrics, such as AUROC, AUPRC, Hit@1, and Hit@10 (detailed in Section 2). SCOPe additionally provides subsets filtered by sequence identity and so permits stress-testing the selected PLMs with increasingly low levels of sequence identity.

Among various important findings, this paper shows that remote homology prediction remains challenging, particularly in the twilight zone, even for small-to-large scale SOTA PLMs. SOTA PLMs experience an average drop of 7.6% in AUROC score when the maximum sequence-identity threshold is lowered from 95% to 10%. This suggests that current PLMs have not sufficiently learned protein structure to address remote homology prediction when sequence signals are weak. We believe the findings in this paper strongly warrant further research both on the problem of remote homology prediction and on the broader goal of learning sequence- and structure-rich representations of protein molecules.

All our evaluation code, benchmark datasets, and models in this paper are made publicly available at https://github.com/amoldwin/plm-zero-shot-remote-homology-evaluation.

## 2 Methods

### 2.1 Problem formulation

We use the classic definition of remote homology and harden it to capture the evolutionary information learned via PLMs. The extended formulation is designed to rigorously test the considered models, enabling us to examine how well structural information is incorporated in the learned sequence representations. We consider a zero-shot approach for identifying remote homologs from learned sequence-representations.

Many recent computational studies for remote homology prediction rely on the hierarchical protein classification system used to annotate proteins in the Structural Classification of Proteins (SCOP2) (Andreeva *et al.* 2013, 2019) and SCOPe (Fox *et al.* 2014, Chandonia *et al.* 2022) databases. In this system, *Family* membership refers to proteins that share a high similarity in their raw sequence but can still exhibit distinct functions. Proteins sharing above 30% sequence identity are generally labeled as belonging to the same Family. On the other hand, two proteins are considered to belong to the same *Superfamily*, which bridges together protein Families, if they share common functional and structural features due to common evolutionary ancestry. The similarity among proteins in a Superfamily is frequently limited to common structural features that, along with a conserved architecture of active or binding sites or similar modes of oligomerization, suggest a probable common evolutionary ancestry. Levels above Superfamily in this protein classification system are identified based on the structural features and similarity. Proteins grouped into structurally similar Superfamilies are labeled to be in the same *Fold*.

#### 2.1.1 Classic definition of remote homologs

We define remote homologs at the Superfamily and Fold levels as in (Chen *et al.* 2018, Strodthoff *et al.* 2020, Rives *et al.* 2021). A pair of proteins *p_i_* and *p_j_* are remote homologs at the Superfamily level if they belong to the same Superfamily but are in different Families [see Equation (1)]. Similarly, a pair of proteins are remote homologs at the Fold level if they belong to the same Fold but are in different Superfamilies.

(1) $$\mathrm{areRemoteHomologs}(p_{i},p_{j})=\{\begin{array}{cc} 1, & \text{if}\;SF_{i}=SF_{j}\;\text{and}\\ & F_{i}\neq F_{j}\\ 0, & \text{otherwise} \end{array}$$

where *SF_i_* and *F_i_* determine the Superfamily and Family label annotation of the *i*th protein.

#### 2.1.2 Hardened definition: remote homology

We harden the classic definitions (whether at the Superfamily or Fold level) to accommodate a sequence identity threshold through which we can gradate the problem and venture into the twilight zone of <30% of sequence identity. The hardened formulation at the Superfamily level is related in Equation (2).

(2) $$\mathrm{areRemoteHomologs}(p_{i},p_{j},th)=\{\begin{array}{cc} 1, & \text{if}\;SF_{i}=SF_{j}\;\text{and}\\ & F_{i}\neq F_{j}\;\text{and}\\ & \mathrm{seqident}(p_{i},p_{j})\leq th\\ 0, & \text{otherwise} \end{array}$$

where *th* denotes the sequence identity threshold that can be decreased to restrict the definition to increasingly hard cases of remote homologs.

### 2.2 Zero-shot remote homology prediction with PLMs

We now describe how to utilize sequence representations of proteins learned from a PLM after pre-training, for remote homology prediction. We refer to this setting as the zero-shot setting.

#### 2.2.1 PLM-learned sequence representations

For each protein, 1≤i≤N, defined by its sequence of *l_i_* amino acids, we obtain the sequence representation $s_{i}\in R^{l_{i}\times D}$ from the last layer of each model. Here, each amino acid is mapped into *D*-dimensional space ($R^{D}$). Next, we compute the protein/sequence-level representation $p_{i}\in R^{1\times D}$ by applying an average pooling layer on the amino-acid level features over the sequence length as in:

(3) $$p_{i}=\frac{1}{l_{i}}\sum_{j=1}^{l_{i}}s_{ij}$$

#### 2.2.2 Comparison of PLM-learned sequence representations

Similarities of high-dimensional vector representations of protein sequences can be compared using distance functions. We adopt the cosine similarity between vector representations from each pair of protein sequences as our similarity metric, following the methodology in (Rives *et al.* 2021). Specifically, for each pair of sequences, we compute the representation similarity as in Equation (4).

(4) $$\text{cos}(p_{i},p_{j})=\frac{p_{i}\cdot p_{j}}{\left||p_{i}||||p_{j}|\right|}$$

#### 2.2.3 Zero-shot remote homology prediction

We define a database *D* of *N* proteins, that is prefiltered such that no proteins share a sequence identity more than a predefined threshold *th*. We use each protein sequence from *D* as an independent query, *q_i_*, against a smaller database *D_i_* where $D_{i}\subset D$. *D_i_* is computed by excluding proteins from *D* based on each *q_i_*. Specifically, we exclude all proteins belonging to either the same Family *F_i_* as the query (when evaluating Superfamily level remote homology) or Superfamily *SF_i_* (when evaluating Fold level remote homology). This exclusion of proteins from the same Family (or Superfamily) as the query enables us to formulate the problem as remote homology versus nonhomology, contrary to remote homology versus “all others,” where “all others” might contain nonremote homologs. We adopt the former formulation in order to understand the models’ true capacity to identify remote homologs rather than their ability to distinguish sequence homologs from remote ones.

In our evaluation, we define remote homologs in accordance with Equation (2) (the hardened definition of remote homology): for a given query, all remote homologs are given a positive label, and all nonhomologs are given a negative label. If there exist no positively labeled target sequences for a query, we remove that query from the evaluation. In this manner, the number of negative labels per query is much higher than the number of positive labels. For instance, the average number of positive labels per query is ∼21 compared with ∼6,756 negative labels at the 10% sequence identity threshold when considering Superfamily-level remote homologs. Similarly, at the Fold level, the average number of positive and negative labels per query are ∼68 and ∼6,692, respectively.

### 2.3 Evaluated models

As related in Section 1, we use seven publicly available, pre-trained SOTA PLMs to obtain representations for our analysis. As a selection of baselines we choose two models, summarized first below.

**Random**: We define random protein sequence representations as sequences of uniformly selected random numbers, each with a length of 150. Then we compute the evaluation metrics based on these randomly initialized protein sequence representations, keeping the ground-truth homology labels from the original sequences. Note that while we observe that these random representations produce slightly different results at each threshold (see Supplementary Material SA.2 for details), we report the average across all thresholds. This baseline is intended to illustrate how much better all of the other models perform when compared with random guesses on each metric.

**HHblits** (Remmert *et al.* 2012): HHblits is a SOTA method for homology prediction based on sequence alignment. We compute match scores between pairs of proteins using the HHblits software package. This involves computing multiple-sequence alignments among sequences in our protein database and training a hidden Markov model to generate profiles that can be compared to each other to obtain match scores between each pair of proteins. Further detail of the protocol is discussed in the Supplementary SA.2.

#### 2.3.1 Selected PLMs

Different types of PLMs have been developed and studied by researchers. Among these, two sets of PLMs, such as sequence-based and sequence-with-structure based (Bepler and Berger 2019, Kabir and Shehu 2022), are particularly popular. Since we delve into the question of how such structural information is learned implicitly by the PLMs, we exclude those that use structural information in the model development. We also focus on transformer-based PLMs, as recent studies suggest their superiority above other models. We select seven SOTA PLMs, which are now summarized below focusing on the pre-training dataset, the model size, and other important model-specific information.

**TAPE-BERT** (Rao *et al.* 2019): TAPE pre-trained three PLMs separately, such as LSTM, Transformer, and dilated residual network (ResNet), on two pre-training objectives: autoregressive and masked language modeling (MLM) tasks. The authors used the Pfam database (Mistry *et al.* 2021), containing ∼31M protein domains. Sequences in Pfam are clustered into evolutionarily related groups called families. A held-out set of families was reserved for testing while the remaining sequences were used for training/validation. We only considered the transformer-based model learned from the MLM objective in our evaluation.

**ProteinBERT** (Brandes *et al.* 2022): The pre-training scheme in ProteinBert combines language modeling with a novel task of Gene Ontology (GO) annotation prediction. ProteinBert was pre-trained on ∼106M proteins derived from UniProtKB/UniRef90 (The UniProt Consortium 2020), covering the entire tree of life. For each protein, the authors extracted its amino-acid sequence and associated GO annotations (according to UniProtKB). The authors considered only the 8943 most frequent GO annotations that occurred at least 100 times in UniRef90. Of the ∼106M UniRef90 proteins, 46M had at least one of the 8,943 considered annotations (with 2.3 annotations per protein, on average across the 46M proteins). Note that the authors removed all input GO annotations altogether for 50% of the processed proteins during training and evaluation to force the model to predict GO annotations from sequence alone. When performing our evaluation, we follow a similar process and only input the unannotated sequence. ProteinBert is considerably smaller and faster than other comparing models, with only ∼16M trainable parameters.

**ESM1b** (Rives *et al.* 2021): We use the ESM1b (esm1b_t33_650M_UR50S), a 33-layer transformer architecture with ∼650M parameters pre-trained with the masked-language-modeling objective on UR50/S. UR50/S represents the high diversity sparse dataset from the UniRef50 (The UniProt Consortium 2020) representative sequences. Note that there are two other pre-training datasets that are used to model the protein sequences with different levels of diversity to study the transformer’s capacity spanning evolutionary diversity: (i) the low-diversity dataset (UR100) uses the UniRef100 representative sequences; (ii) the high-diversity dense dataset (UR50/D) samples the UniRef100 sequences evenly across the UniRef50 clusters. ESM1b is reported to be the most powerful in the ESM-1 suite of models (Rives *et al.* 2021).

**ESM2** (Lin *et al.* 2022): ESM2 is a new-generation BERT style encoder-only transformer model, trained over millions of sequences on the UniRef protein sequence database. The ESM2 models are trained with the MLM objective. A family of ESM2 models are available at scale from 8 million parameters up to 15 billion parameters. The 33-layer ESM2 model of 650M parameters we select (esm2_t33_650M_UR50D) is a representative of the top three reported models (of size 650M, 3B, and 15B parameters) in the ESM2 suite (Lin *et al.* 2022). While ESM1b used learned positional encodings instead of static sinusoidal positional encodings, ESM2 models used Rotary Position Embeddings (RoPE) to allow the model to extrapolate beyond the context window it is trained on. Another distinction in the ESM2 pre-training is that the training sequences are sampled with even weighting across ∼43 million UniRef50 training clusters from ∼138 million UniRef90 sequences so that over the course of training the model sees ∼65 million unique sequences.

**Prottrans BERT, Albert, and T5** (Elnaggar *et al.* 2022): Prottrans-BERT-BFD is a ∼420M parameters BERT-based transformer encoder model of 30-layers self-attention blocks with 16 attention heads. It limits sequence length context to ∼40K amino acids. Prottrans-Albert-BFD follows Albert’s reduced complexity on BERT by hard parameter sharing between its attention layers which allows it to increase the number of attention heads to 64 compared to Prottrans BERT’s 16. Both models use the Big Fantastic Database (BFD) (Steinegger *et al.* 2019, Steinegger and Söding 2018) merged with UniProt and proteins translated from multiple metagenomic sequencing projects, making it the largest collection of protein sequences available at the time of writing even after removal of duplicates from the original BFD containing ∼393 billion tokens. T5 contains two variants at scaling the number of parameters of ∼3B (Prottrans-T5-XL) and ∼11B (Prottrans-T5-XXL). We choose the smaller version for the model size constraint which is pre-trained on the BFD (Steinegger *et al.* 2019, Steinegger and Söding 2018) dataset. T5 allows reconstructing spans of tokens instead of single tokens. However, contrary to the original T5 model which masks spans of multiple tokens, Prottrans-T5 adopted BERT’s denoising objective to corrupt and reconstruct single tokens using a masking probability of 15%.

### 2.4 Datasets

To provide a comprehensive description of the structural and evolutionary relationships between all proteins whose structure is known, the SCOP (Andreeva *et al.* 2013, 2019, Fox *et al.* 2014, Chandonia *et al.* 2022) provides a database of all known protein Folds, with detailed information about the close relatives of each protein in the database. In this study, we leverage both manually curated SCOP2 (Andreeva *et al.* 2013, 2019) dataset and its extension SCOPe 2.08 (Fox *et al.* 2014, Chandonia *et al.* 2022) that is created using automated tools to help with annotation and error removal.

We download Astral (Brenner *et al.* 2000, Chandonia *et al.* 2002, 2004) domain subsets based on protein sequence percentage identity from SCOPe database (https://scop.berkeley.edu/astral/subsets/ver=2.08). Particularly, we utilize the sequence subsets at of 10%, 20%, 30%, 40%, 70%, and 95% sequence identity thresholds. This wide range of thresholds enables us to understand the variations and discrepancies among PLMs in their capacity for remote homology identification in and outside the twilight zone.

Since pre-computed database subsets at different sequence identity levels are not readily available for SCOP2 (https://scop2.mrc-lmb.cam.ac.uk/download), we apply the widely used CD-HIT (Fu *et al.* 2012) clustering program to filter the SCOP2 database at each identity threshold. Running CD-HIT at 95% and 70% thresholds without changing any other default parameters, yields 27 572 and 23 006 clusters, respectively, and returns representative sequences for each cluster. Next, following CD-HIT’s recommendations, we applied PSI-CD-HIT (Fu *et al.* 2012), which utilizes blast-based sequence identity computation, at the rest of the similarity thresholds.

We carry out minimal data pre-processing, so that future developments in remote homology prediction can be easily validated by repeating our evaluation procedure. Firstly, we remove specific types of proteins such as Rossman-like Folds and four- to eight-bladed *β*-propellers. One other filtering step that we applied was to only include sequences that represent a single continuous span of the underlying protein structure. For SCOP2 derived datasets, this same span must denote the Superfamily and Family, otherwise we exclude the sequence from our evaluations. Finally, if a SCOPe sequence has a concise classification string (sccs) representation that also considers subdomains, that are not Class, Fold, Superfamily or Family, we exclude that sequence. More details of the data preprocessing steps and CD-HIT usage can be found in the Supplementary Section SA.1.

Supplementary Table S3 summarizes the dataset statistics at different sequence percentage identity thresholds for SCOPe and SCOP2 with counts of the datapoints, Folds, Superfamilies and Families. The number of datapoints for SCOPe derived datasets decreases from 33,771 to 6,784 for increasingly difficult thresholds of 95% to 10% sequence identity, respectively. The statistics also demonstrates that we lose relatively few classes, i.e. Folds, Superfamilies, and Families, due to the minimal data-processing steps.

### 2.5 Performance metrics

We compute several metrics per query: the area under the receiver operating characteristic curve (AUROC), area under the precision-recall curve (AUPRC), Hit@1, and Hit@10. All of these metrics operate over the cosine similarity between two (PLM-learned) sequence-level representations, the representation of a query sequence and that of another sequence.

Consider a query sequence *QS*. We compute the cosine similarity between the representation of *QS* and the representation of all other sequences in the database. Hit@1 and Hit@10 rely on sorting all the cosine similarity scores in a descending order. In Hit@1, the attention is on the top score. If the sequence corresponding to the top score is labeled positive (i.e. remote homologous, as described in Zero-shot Remote Homology prediction), the Hit@1 score for the given query is 1; 0 otherwise. Averaging over different query sequences (obtained over the database) provides us with a Hit@1 score over the entire distribution. The calculation of Hit@10 follows a similar process, but the attention is on the top ten hits (the top ten similarity scores); if any of the hits corresponds to a positively labeled sequence, the Hit@10 score is 1; 0 otherwise.

The AUROC and AUPRC rely on a classification threshold which is varied between 0 and 1. In our case, the threshold is based on the cosine similarity. All sequences with cosine similarity score no higher than a given threshold are considered positives with the others considered negatives. Comparison with the actual labels of the sequences (again assigned as described in Zero-shot Remote Homology prediction) provides us with true positives (TP), true negatives (TN), false positives (FP), and false negatives (FN). Based on these, one can then calculate the True Positive Rate (TPR) as in $\text{TPR}=\frac{\text{TP}}{\text{TP}+\text{FN}}$ and False Positive Rate (FPR) as $\text{FPR}=\frac{\text{FP}}{\text{FP}+\text{TN}}$. Different values of TPR in response to the moving threshold provides us with the receiver operating characteristic curve (ROC) and the corresponding area under the ROC, the AUROC. The precision versus recall curve (PRC) relates the precision (calculated as $\text{Precision}=\frac{\text{TP}}{\text{TP}+\text{FP}}$) versus the recall (this is the same as TPR) as one varies the threshold. AUPRC measures the area under the PRC.

We report the weighted performance measurements as our primary findings, where we first compute the averaged metrics per Superfamily or per Fold, and then compute the averaged performance following the practices defined in Söding and Remmert (2011). This helps facilitate a more holistic evaluation that takes advantage of the diversity of proteins present in SCOPe and SCOP2 by ensuring that smaller superfamilies and Folds affect the reported metrics as much as large ones. We also report the nonweighted averaged performance across all queries in the Supplementary Section SA.3 as in Rives *et al.* (2021).

This choice in metrics relates to the trade-off between recall and precision, which is influenced by whether the user will be more inconvenienced by false negatives (“Type 2” errors) or false positives (“Type 1” errors) in the model’s predictions. The AUROC and AUPRC scores are based on True/False positive rates, and precision/recall scores respectively, while the “hit” scores place high importance on not having a long list of false positives at the top of the ranking.

Both Type 1 and Type 2 errors are significant. FPs are important to avoid because researchers trying to identify previously unknown evolutionary relationships may be concerned with proteins where finding evidence to validate a model’s homology prediction will often be difficult. In such cases, sorting through a long list of FPs before arriving at the first TP may be impractical. FNs, on the other hand, will mean that important discoveries could be missed because the most difficult-to-identify homologs may have the most to reveal about nontrivial evolutionary and functional relationships between proteins.

In addition to the per-model metrics described above, we also use Spearman’s correlation coefficient to assess Superfamily-level agreement in performance between each pair of models that we test. Let *L_i_* and *L_j_* denote the lists of AUROC scores achieved by models *i* and *j* on each Superfamily, respectively. We calculate Spearman’s rank correlation coefficient between *L_i_* and *L_j_* as $\rho_{L_{i},L_{j}}=1-\frac{6\sum_{k=1}^{n}{\left(r_{L_{i},k}-r_{L_{j},k}\right)}^{2}}{n(n^{2}-1)}$, where $r_{L_{i},k}$ and $r_{L_{j},k}$ are the ranks of the *k*th scores in *L_i_* and *L_j_*, respectively, and *n* is the number of Superfamilies. This coefficient quantifies the agreement between the two models regarding which Superfamilies were “easy” or “difficult” to detect homology in. We additionally compute this using lists of per-query AUROC performance and also for Fold-level remote homology, using Fold-level lists of AUROC scores.

## 3 Results and discussion

**Figure 1.**
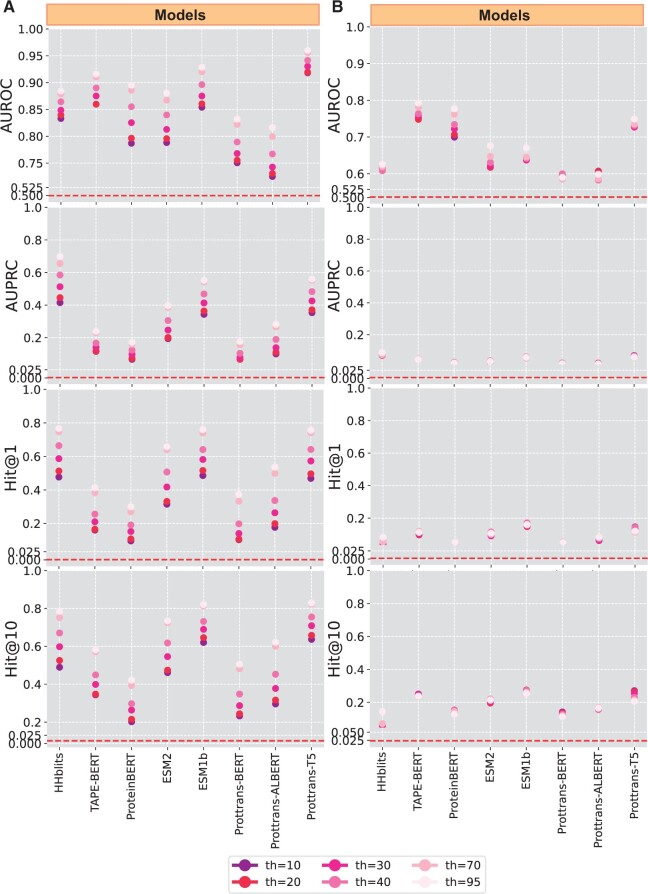
Models are evaluated on the SCOPe dataset at the Superfamily-level formulation according to the AUROC, AUPRC, Hit@1, and Hit@10 performance metrics. Shading indicates different thresholds of sequence identity, with darker shades indicating lowering identity. Panel (A) relates findings at the Superfamily level, and panel (B) does so at the Fold level. The performance of a random model, as described in Section 2 is shown through the dotted line.

**Figure 2.**
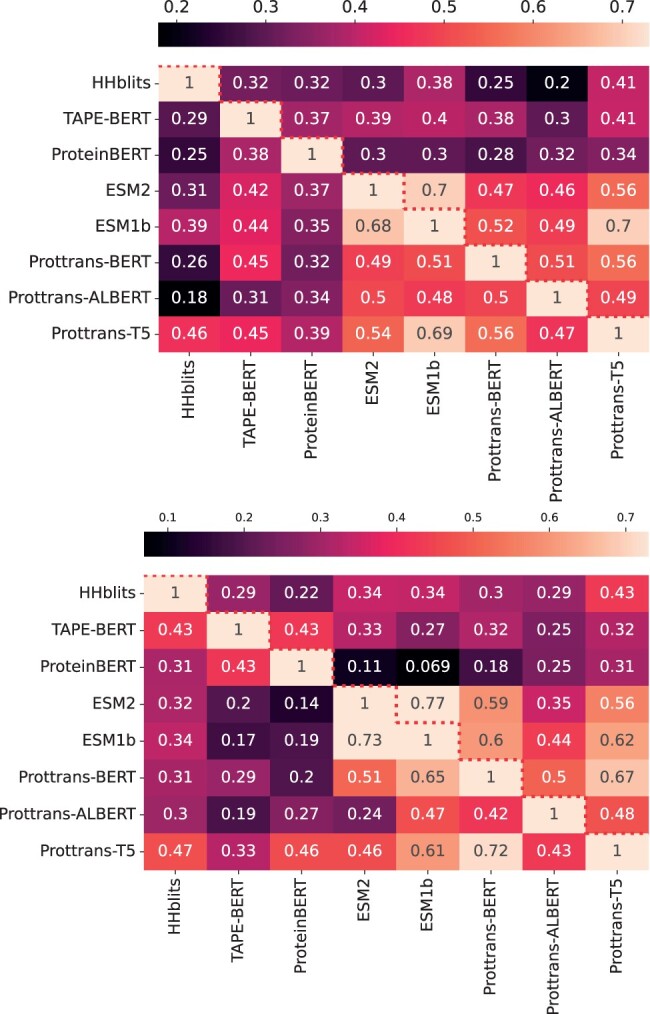
(A) Top panel compares the AUROC scores (via Spearman’s rank correlation coefficient) between pairs of models at 30% sequence identity at the per-Superfamily level below the dotted line and at the per-query level above the dotted line. (B) Bottom panel does so at the Fold level.

We present three sets of results. First, in Fig. 1 we relate the comparative dataset-wide performance of the various models on AUROC, AUPRC, Hit@1, and Hit@10 at both the Superfamily and Fold levels of remote homology at decreasing sequence identity. The second set of results in Fig. 2 quantifies the relative agreement between the performance of the models, focusing on the AUROC metric. In the third set of results, related in Tables 1 and 2, we focus on specific Superfamilies that highlight aspects of remote homology prediction that are trivial versus challenging.

**Table 1.** Top panel: Superfamilies where Prottrans-T5 achieves perfect AUROC when the maximum-identity threshold is set to 30%; Bottom panel: The five Superfamilies with the lowest AUROC at the 30% threshold using Prottrans-T5.^a^

SF	Description	AUC	#-True	#-QSs
**Top**				
a.38.1	HLH, helix-loop-helix DNA-binding domain	1.00	1.71	7
a.39.2	Insect pheromone/odorant-binding proteins	1.00	4.44	9
a.64.1	Saposin	1.00	4.67	6
a.87.1	DBL homology domain (DH-domain)	1.00	1.71	7
b.22.1	TNF-like	1.00	1.80	10
b.74.1	Carbonic anhydrase	1.00	1.67	6
c.44.1	Phosphotyrosine protein phosphatases I	1.00	2.67	6
c.54.1	PTS system fructose IIA component-like	1.00	4.00	7
d.189.1	PX domain	1.00	5.45	11
d.95.2	Homing endonucleases	1.00	5.33	12
e.1.1	Serpins	1.00	6.15	13
**Bottom**				
d.145.1	FAD-binding/transporter-associated domain-like	0.56	13.44	18
b.52.1	Barwin-like endoglucanases	0.59	5.14	7
a.6.1	Putative DNA-binding domain	0.61	11.08	13
d.224.1	SufE/NifU	0.63	3.14	7
a.60.8	HRDC-like	0.64	7.40	10

a *Top panel*: This list is filtered to only show Superfamilies with more than five queries available at this threshold setting. The “SF” column shows the “scc” identifier for the Superfamily in the SCOPe database. The “#-True” column shows the average number of true labels per query, and the “#-QSs” column shows the number of available queries for each Superfamily at the 30% threshold. Note that the number of negative labels for each of these is 10 360±30. *Bottom panel*: As in the top panel, the list is filtered to only show Superfamilies where more than five query sequences are available.

**Table 2.** Top panel: Superfamilies with the highest difference in average AUROC between Prottrans-T5 and HHblits at the 30% threshold, showing the Superfamilies where Prottrans-T5 achieved significantly higher AUROC than HHblits; Bottom panel: Superfamilies with the highest difference in average AUROC between HHblits and Prottrans-T5 at the 30% threshold, showing the Superfamilies where HHblits performed better than Prottrans-T5.

SF	Description	HHblits AUC	T5 AUC
**Top**			
d.58.17	HMA, heavy metal-associated domain	0.50	1.00
d.58.3	Protease propeptides/inhibitors	0.50	1.00
d.58.36	Nitrite/Sulfite reductase N-terminal domain-like	0.50	1.00
d.58.32	FAD-linked oxidases, C-terminal domain	0.50	1.00
d.21.1	Diaminopimelate epimerase-like	0.50	1.00
d.42.1	POZ domain	0.50	1.00
d.15.4	2Fe-2S ferredoxin-like	0.50	1.00
d.37.1	CBS-domain pair	0.50	1.00
d.15.2	CAD & PB1 domains	0.50	1.00
a.61.1	Retroviral matrix proteins	0.42	0.96
**Bottom**			
c.114.1	DsrEFH-like	0.99	0.83
a.130.1	Chorismate mutase II	0.99	0.79
c.97.3	JAB1/MPN domain	0.98	0.66
b.52.2	ADC-like	0.97	0.73
d.79.3	L30e-like	0.94	0.69
a.60.8	HRDC-like	0.87	0.64
c.26.2	Adenine nucleotide alpha hydrolases-like	0.87	0.67
d.224.1	SufE/NifU	0.83	0.63
a.6.1	Putative DNA-binding domain	0.79	0.61
d.145.1	FAD-binding/transporter-associated domain-like	0.77	0.56

Figure 1 shows the performance of each model on each of the performance metrics on the SCOPe database at the Superfamily level in Panel A and at the Fold level in Panel B. Color-coding tracks the different sequence identity thresholds, with darker shades denoting increased difficulty (lower sequence identity from 95% to 10%). The Supplementary Material relate these results in a tabular format in Supplementary Tables S5 and S6.

Figure 2 visualizes the Spearman’s rank correlation coefficient when comparing pairs of models on AUROC scores obtained at 30% sequence identity. The bottom left triangle (below the dotted red line) in the top panel focuses on the per-Superfamily AUROC scores averaged over the Superfamilies. The upper right triangle (above the dotted red line) focuses on the per-query AUROC scores averaged over all the queries. The bottom panel does so at the Fold level.

As our discussion will later show, the model with consistently top performance among the PLMs across the various settings that we investigate is Prottrans-T5. Using the performance of this model as a guide, we narrow our attention in Table 1 to the Superfamilies where Prottrans-T5 achieves the highest (top panel) and the lowest (bottom panel) AUROC, respectively, at the 30% threshold. For these Superfamilies we show the average number of true labels per query and the number of available queries. In Table 2, we broaden our attention to include the Superfamily level performance of HHblits, the non-PLM that represents sequence alignment-based methods, and show the Superfamilies that elicit the biggest difference in performance in terms of AUROC between Prottrans-T5 and HHblits. The top panel of Table 2 considers the setting of Prottrans-T5 AUROC minus HHblits AUROC, and the bottom panel considers the opposite, HHblits AUROC minus Prottrans-T5 AUROC.

As we will describe in greater detail below, the three sets of results related above support the following main observations:

- As sequence identity decreases, the performance of all the models, including the PLMs, deteriorates.
- Where PLMs exhibit low performance, they do so for different reasons. We observe low agreement on which Superfamilies are difficult across the PLMs.
- PLMs achieve comparable performance to HHblits.
- The ESM suite of PLMs exhibits surprising behavior. In particular, ESM2 is outperformed by ESM1b across all metrics of performance.
- The manually curated dataset, SCOP2, is more challenging for all models than the computationally extended SCOPe.

We now focus our discussion on each of these observations. The Supplementary Materials provide further evidence that support our main findings.

### 3.1 As difficulty increases, performance deteriorates

Let us first focus on our findings at the Superfamily level (Fig. 1A). While some models perform better than others regardless of the threshold, each model exhibits a diminished performance as the threshold is lowered. For instance, the largest difference in AUROC, when dropping from 95% to 10% sequence identity, occurs for ProteinBERT, where the score drops from 90% to 79% as the threshold is lowered. Prottrans-T5 is impacted least of the others; its AUROC score drops from 96% to 92% as the threshold drops from 95% to 10% sequence identity. The average drop in AUROC across all eight models is 7.6%.

The extent of the divergence in performance varies among metrics and models. Specifically, for Hit@1, where the first nearest neighbor of the query sequence in the representational space is considered as the positive sample, the changes in performance are substantial. For example, ESM1b, which achieves 76% Hit@1 at the 95% threshold on the SCOPe dataset at the Superfamily level, drops to 49% and 52% at the 10% and 20% thresholds, respectively.

Hit@10 scores offer a slightly easier setting, as it allows exploring the vicinity of a given query sequence in the representational space. Most PLM models perform significantly better under this relaxed metric. For instance, ESM1b’s scores at the 10% and 20% sequence identity thresholds rise to 62% and 65%, respectively. Interestingly, the HHblits baseline performs almost identically under Hit@10 and Hit@1 (i.e. only a 1% difference in scores at many thresholds), indicating that when hits are achieved by HHblits, they are likely to be ranked first to begin with.

In contrast to the Hit metrics, AUROC scores consider the quality of the entire ranking of target sequences in relation to the query and show a slightly less pronounced change due to the reduced threshold. Due to the extreme infrequency of the positive class in our dataset, AUROC scores can remain deceptively close to 1 and fail to appropriately differentiate between good and bad performance. This can be remedied by considering AUPRC. As the AUPRC scores are predictably much lower than AUROC (Supplementary Table S5 for quantitative values) and show a much more dramatic decrease in performance as the threshold is lowered. While the best performance in AUROC is achieved by Prottrans-T5, this model drops to second place in AUPRC, with AUPRC scores dropping from 56% to 35% as the threshold drops from 95% to 10% sequence identity.

This consistent loss in performance at lower thresholds, particularly below 40% sequence identity, for all models and across all metrics, is an indication that homology prediction remains difficult even for the SOTA models. We note that at the Fold level (Panel B), we observe consistent poor performance of all models at identifying remote homologs. While three out of seven PLMs marginally surpass the 20% threshold in Hit@10, none of the models achieve such performance in Hit@1.

### 3.2 PLMs exhibit low agreement on which superfamilies are difficult

We now turn our attention to the models’ comparative performance on individual Superfamilies (Fig. 2, top panel). This reveals that not all domains of proteins are similarly difficult or easy for each PLM, even when considering PLMs with comparable average AUROC performance. For example, while Fig. 1A shows ProteinBERT and ESM2 achieving similar AUROC scores across all thresholds, ESM2 shares a relatively low 37% per-Superfamily correlation with ProteinBERT, and a higher 68% correlation with ESM1b in the top panel of Fig. 2, below the dotted line. These differences seem to indicate that the model type and pre-training data play a key role in determining which cases of remote homology will be difficult to identify. This may also indicate that ensemble methods may be useful to exploit the strengths of multiple model types.

The region below the dotted line in Panel B of Fig. 2 similarly shows Spearman’s rank correlation coefficient for per-Fold AUROC scores between each pair of models. In this, the pairs of models that exhibit high agreement tend to be similar to those in Panel A, and again do not fully correspond with models having similar average AUROC performance in Fig. 1. The three models with highest AUROC at the Fold level in Panel B of Fig. 1 were TAPE-BERT, ProteinBERT, and Prottrans-T5, but the highest correlations at the Fold level were instead observed between ESM1b and ESM2 (73%), and between Prottrans-T5 and Prottrans-BERT (72%), indicating that the choice of pre-training datasets and model-type is an important factor in determining which Folds are easier or harder to identify.

### 3.3 PLMs achieve comparable performance to HHblits

It is worth expanding more on the findings related in Fig. 1A (on SCOPe at the Superfamily level) regarding the performance of HHblits in comparison to the PLMs.

We observe that HHblits achieves the highest score across all thresholds when considering AUPRC, with an average improvement of 11.6% over the next-best model, Prottrans-T5, and shows even greater advantages when compared with ESM1b; Supplementary Table S5 provides tabular data. However, HHblits shows poorer performance according to AUROC and Hit@10 when compared with the same PLMs. (Note that in contrast to our results, HHblits achieves superior performance to PLMs in (Rives *et al.* 2021) when considering both of these metrics; this is likely due to a higher number of iterations when performing the multiple sequence alignments for HHblits in their study.)

HHblits additionally differed from PLMs when considering its performance on individual Superfamilies. The bottom triangle of Fig. 2A shows that HHblits AUROC scores have low correlation with those of any of the other PLMs (the highest being 46% correlation with Prottrans-T5); most of the high-performing PLMs show higher correlations with each other (e.g. ESM1b and Prottrans-T5 share a 69% correlation). When examining the per-Fold correlation analysis depicted in the lower triangle of Fig. 2B, we observe that despite HHblits having a moderately high correlation of 47% with Prottrans-T5, Prottrans-T5 is more strongly correlated with ESM1b and Prottrans-ALBERT, with correlation coefficients of 61% and 72%, respectively. At the per-query level, the upper triangles in Fig. 2A and B show darker shades across the top row, indicating low correlation between PLMs and HHblits.

Because HHblits is a drastically different method for identifying remote homology when compared with PLMs, the strengths and weaknesses of each method are of particular interest. The top panel of Table 2 indicates that several Superfamilies exhibit perfect performance when using Prottrans-T5, while HHblits is no better than a random predictor (50% AUC) for the same Superfamilies. This implies that at least in some cases PLMs are learning aspects of remote homology that are not accounted for at all in HHblits. Taking “retroviral matrix proteins” as an example, we note that biologists have observed this to be a Superfamily where the association between its proteins is often evidenced by physical features that cannot be predicted by any specific sequence motif (Murray *et al.* 2005).

To a somewhat lesser degree, the bottom panel of Table 2 shows that there are similarly Superfamilies where HHblits identifies remote homology nearly perfectly, while Prottrans-T5’s performance is significantly worse. Looking at the entry “JAB1/MPN domain,” we see an example of a Superfamily that is associated with a specific motif (Ambroggio *et al.* 2004), helping explain why HHblits is effective for this Superfamily. We speculate that PLMs struggle with this Superfamily due to other unknown structural factors that cause the representations for the sequences in this Superfamily to be far apart from each other. This highlights a possible pitfall of using PLM representations for remote homology prediction: they can fail even in cases when sequence information alone should provide evidence of homology. This also may relate to observations in (Kilinc *et al.* 2022) that PLM representations are more suited to global homology detection and may fall short when the sequence similarity is localized to a small fragment of the sequences.

### 3.4 Prottrans-T5 demonstrates superior performance on AUROC

Prottrans-T5 exhibits superior performance at the Superfamily level, particularly when considering low sequence identity. Across all sequence identity thresholds, Prottrans-T5 achieves AUROC scores above 90%, with the lowest score of 92% at 10% identity and a maximum score of 96% at 95% identity. In comparison, its nearest competitor, ESM1b, achieves scores of 85% and 93% at the same thresholds, respectively, with a standard deviation of 0.032 compared to Prottrans-T5’s 0.018. Notably, TAPE-BERT exhibits less variation (standard deviation of 0.025) than ESM1b but performs worse at higher thresholds.

Several factors may contribute to Prottrans-T5’s superior performance. First, larger neural network models with millions to billions of parameters tend to perform better on downstream tasks when pre-trained on extensive datasets. This is especially true in zero-shot settings like ours, where remote homologs are predicted based on their proximity in the models’ representational space without any task-specific fine-tuning. In such cases, the size of the model and breadth of the pre-training datasets can play a more significant role in performance than small variations in neural network architecture. This also explains why many PLMs adopt architectures and training objectives originally designed for natural language processing without suffering from decreased performance. The large models and extensive training data provide the necessary capacity and information to effectively learn and predict protein relationships, even without specialized architectural adjustments.

When considering the effect of model size on the performance of each model, we note that the smallest models, ProteinBert (∼16M parameters) and TAPE-BERT (∼38M parameters), performed relatively poorly. This is despite Protein Bert’s inclusion of the Gene Ontology (GO) prediction task in its pre-training, indicating that additional training tasks may not be sufficient to compensate for a lower number of parameters. The next-smallest models, from lowest to highest number of parameters, were Prottrans-ALBERT (∼240M), Prottrans-BERT (∼420M), ESM1b (∼650M), ESM2 (∼650M), and finally Prottrans-T5 (∼11B). While the gain in performance exhibited by these models does not directly correlate with model size, larger models do tend to outperform the smaller ones when considering AUPRC and Hit scores (e.g. TAPE-BERT marginally exceeds Prottrans BERT’s scores at certain thresholds). This parameter-to-performance scaling is particularly important in practice because while most of the medium-size models shown here can fit on a consumer GPU, the larger models such as T5 and even the ESM models are not always usable without heavy-duty, production GPUs. This may preclude some researchers from using the larger models, limiting the performance that will be practically available for PLM-based remote homology detection in ordinary research settings.

### 3.5 ESM2 is outperformed by ESM1b across all metrics

We observe that, despite ESM2 being a more “updated” model that performs better than ESM1b on other downstream tasks, its Superfamily-level remote homology performance shown in Fig. 1A was consistently worse than that of ESM1b, across all four metrics. Averaging across all identity thresholds, ESM2 achieved AUROC scores 6% lower and AUPRC scores that were 16% lower than those of ESM1.

Because we consider comparably sized ESM-1 and ESM-2 models, the variance in the learned representational space’s capacity may stem from disparities in the pre-training datasets. Specifically, ESM1b is pre-trained on the high-diversity sparse dataset (UR50/S), consisting of UniRef50 (Suzek *et al.* 2015) representative sequences clustered at 50% sequence identity, whereas ESM2 utilizes the high-diversity dense dataset (UR50/D), sampled evenly from the UniRef100 sequences across the UniRef50 clusters. It is conceivable that the protein sequences within the sparse dataset serve as better representatives across protein Families and Superfamilies, thereby enabling the model to acquire more effective representation space, respective to this particular task.

### 3.6 Manually curated datasets are more challenging

Our evaluation of PLMs on SCOP2, with results shown in Supplementary Fig. S4, demonstrates that datasets derived from SCOP2 present significant challenges for PLMs compared to SCOPe (whose results we relate above in Fig. 1). For example, Prottrans-T5 achieves an AUROC score of 96% in SCOPe-based Superfamily-level remote homology detection, whereas its performance drops to 92% for datasets derived from SCOP2 at the same threshold. Similarly, ESM1b’s performance significantly decreases from 93% to 84% at high sequence identity levels. Overall, the performance of all models is more severely impacted at high sequence identity levels than it is at low sequence similarity. The phenomenon that we observed with SCOPe, where remote homology detection problem became increasingly challenging as sequence similarity decreased, is not as pronounced in SCOP2 datasets. Nevertheless, the SCOP2 dataset consistently presents significant difficulty across all sequence similarity thresholds for all models. Similar to the SCOPe Fold-level remote homology detection, SCOP2 poses significant challenges for PLMs. None of the models achieve a Hit@1 score above 13% at any sequence identity threshold. Similarly, no model exceeds a Hit@10 score of 24%. In contrast, for SCOPe-based Fold-level remote homology detection, the corresponding scores were 17% and 28%, respectively.

## 4 Conclusion

In this study, we have explored the capacity of transformer-based PLMs trained over protein sequence data to implicitly learn structural information. We have selected a hallmark problem in computational biology, remote homology prediction tasks, to do so. The problem becomes increasingly difficult as one enters the twilight zone of sequence homology, where remote homologs can be found that have <30% sequence identity. In the twilight zone, as one cannot rely on sequence identity, correctly identifying shared structural features is key for identifying remote homologs.

To stress test sequence-trained PLMs, we harden the problem formulation of remote homology prediction to include sequence identity and gradate it over decreasing sequence identity, so we can build performance profiles of PLMs as they enter the twilight zone. Through rigorous evaluation across a range of identity thresholds, we elucidate the abilities of current PLMs to detect remote homologs at both the Superfamily and Fold levels in the zero-shot setting; i.e. using representations obtained right after pre-training with no fine-tuning on any particular downstream prediction tasks.

Our experiments show that when assessing Superfamily level homology prediction, the performance of PLMs consistently deteriorates when the maximum-allowed sequence identity shared by a pair of proteins is decreased from 90% to 10%. We observe a noticeable decline in standard metrics of performance, such as AUROC, AUPRC, Hit@1, and Hit@10 scores as sequence identity thresholds are lowered. These decreases in performance are consistent with biological theory and reflect the challenges of identifying structural and functional similarities between proteins when sequence identity is low. While PLMs have proven effective for certain kinds of remote homology prediction, the limitations revealed here are important to be aware of in order to effectively utilize these tools.

Our comparison of different PLMs yields many insights, including several unexpected results. Prottrans-T5 demonstrates overall superior performance among the considered PLMs on both the SCOPe and SCOP2 derived remote homology datasets, and even exceeds the performance of the alignment-based HHblits model on certain metrics. Additionally, ESM1b overall outperforms its updated sister-model ESM2 in this setting. The sequence alignment-based model HHblits is consistently a better performer at the Superfamily level remote homology identification when compared to the other PLMs, such as TAPE-BERT, ProteinBERT, Prottrans-BERT and Prottrans-ALBERT. Fold level remote homology prediction remains highly challenging in and outside of the twilight zone. We also observe that models with similar design and pre-training objectives are most likely to show high agreement regarding the least and most challenging Superfamilies or Folds for remote homology prediction. This remains true even for models whose overall performance differs markedly when averaging across Superfamilies or Folds, highlighting the diversity in the types of structural information needed to fully model all Superfamilies.

Our results additionally show that PLMs can sometimes fail even in cases when sequence information alone ought to provide sufficient evidence of homology. This suggests that PLM-learned representations are currently better able to leverage global rather than local sequence similarity, potentially opening a new avenue for future research on better PLMs for remote homology prediction. In this way, the strengths exhibited by future PLMs may complement those of alignment-based methods and be useful in applications such as antibody engineering where alignment-based methods have often been insufficient.

Taken altogether, our findings support the conclusion that current PLMs have not sufficiently learned protein structure to address remote homology prediction but do exhibit certain strengths when compared with alignment-based methods.

These findings strongly warrant further research both on the specific problem of remote homology prediction and on the broader computational biology objective of learning sequence- and structure-rich representations of protein molecules.

## Supplementary Material

vbae119_Supplementary_Data

## Data Availability

All code, data, and models in this paper are made publicly available at https://github.com/amoldwin/plm-zero-shot-remote-homology-evaluation.
