# Supplementary material for: In the twilight zone of protein sequence homology: do protein language models learn protein structure?
Source: Bioinform Adv. 2024 Aug 17;4(1):vbae119. doi: 10.1093/bioadv/vbae119 (PMC11344590; doi:10.1093/bioadv/vbae119)
Supplement: vbae119_Supplementary_Data [file vbae119_supplementary_data.pdf]

Supplementary Materials

Here we provide details on our data pre-processing steps, baseline models, and extended results.

Details of Data Preprocessing and Dataset Statistics

Data preprocessing: We remove specific types of proteins such as Rossman-like Folds and four- to eight-bladed β-propellers in our data preprocessing steps. In SCOPe, these Folds are labeled by c.2-c.5, c.27, c.28, c.30 and c.31 (Rossman Fold), and b.66-b.70 (4-to-8 bladed β-propellers) as in (Rives et al., 2021a). In SCOP2, these are denoted by Canonical, SDR-type extended, OCD-type extended, Variant, CoA-binding domain-type and 6PGDH-type extended Rossmann Folds, and 4- to 8-bladed β-propellers. These domains are removed following standard practices (Söding and Remmert, 2011) because, while members from different Fold domains are generally considered non-homologous, the status of this set of Folds is not known.

About CD-HIT usage: By default, CD-HIT places each sequence in the first cluster that meets the similarity threshold, which is faster but less-accurate than ensuring that each sequence will be placed into the closest-possible cluster. For this study, we chose the latter, more accurate option. PSI-CD-HIT also reports the representatives of final clusters. Since these greedy-heuristic based cluster representatives do not depend on the clustering mechanism, we randomly select one representative per cluster. We evaluate and report the performance of the considered PLMs on both CD-HIT representatives and randomly selected representative sequences.

Dataset Statistics: Table 3 summarizes the dataset statistics at different sequence percentage identity thresholds for SCOPe and SCOP2 with counts of the datapoints, Folds, Superfamilies and Families. This also demonstrates that we lose relatively few datapoints because of the minimal data-processing steps.

Threshold	Datapoints	Folds	Super-families	Families
		**SCOPe**		
10	6,784	1,220	1,985	3,999
20	7,547	1,229	1,995	4,116
30	10,370	1,229	1,997	4,310
40	14,280	1,230	1,998	4,521
70	23,349	1,230	1,998	4,976
95	33,771	1,230	1,998	4,826
		**SCOP2**		
10	9,852	1,488	2,589	5,123
20	10,074	1,488	2,589	5,127
30	11,878	1,488	2,589	5,131
40	14,380	1,448	2,407	4,881
70	23,066	1,489	2,589	5,135
95	27,572	1,489	2,589	5,135

Table 3. Below are the data statistics for SCOPe- and SCOP2-derived datasets following preprocessing steps at various sequence percentage identity thresholds. Note that for SCOP2 an increased threshold does not always equate to an increase in the number of Superfamilies or Folds because the clusters were re-evaluated for each threshold.

Further Model Details

Here we provide more details for each baseline.

Random: Since the differences across thresholds are small for the random baseline and the focus is to see whether the PLMs can effectively outperform the random baseline, which they do, we report the average performance metrics across all considered sequence identity thresholds, denoted as the red dotted line in Figs. 1 (A) and (B) . The actual values for each threshold are shown in Table 4.

Threshold	AUROC	AUPRC	Hit@1	Hit@10
10	0.495	0.001	0.001	0.010
20	0.498	0.001	0.001	0.008
30	0.505	0.001	0.001	0.009
40	0.501	0.001	0.001	0.007
70	0.499	0.001	0.001	0.007
95	0.499	0.001	0.001	0.007

Table 4. Metrics obtained for Superfamily-level remote homology using the random-embedding baseline.

HHblits: We compute match scores between pairs of proteins using the HHblits software package. This involved using the HH-Suite software to compute multiple-sequence alignments between sequences in our protein database, and training hidden Markov model "profiles" that can be compared to each other to obtain match scores between each pair of proteins. For each of these steps, we used the default recommended settings from the HH-Suite software package, with the only deviation being our imposition of a maximum-memory limit of $3.4GB$ per process when computing the Multiple-sequence alignment. When performing the ranking of potential matches, we rely on the "E-Value" rather than the probability score, as in (Rives et al., 2021a). Note that even better performance has been observed in Rives et al. (Rives et al., 2021a) by increasing the number of iterations from 2 to 3 when building the multi-sequence-alignment. Due to resource constraints, we retained the default value of 2 for the purposes of this study.

Effect of Weighting Results by Superfamily

Comparison of eighted versus unweighted performance scores across Superfamily or Fold classes reveals minimal deviations. Previously, Söding and Remmert (Söding and Remmert, 2011) discussed that the number of homologous pairs scales as the number of members squared. Consequently, large Superfamilies would have a dominant influence on the AUROC analysis. Following this standard practice, we compare weighted versus unweighted performance metrics across Fold and Superfamily level remote homology detection tasks. In Figure 3, we present the unweighted results for Superfamily (left panel) and Fold (right panel) level remote homology detection. Comparing with weighted Superfamily and Fold (Figure 1 (A) and (B) (top-left panel), respectively), we find minimal impact of applying weights. However, we consider the weighted results to be our main findings in this manuscript because applying weights effectively mitigates bias towards classes with a large number of examples.

SCOP2 Results

Here we report the SCOP2-based models' performance. Figure 4 demonstrates overall performance comparison among

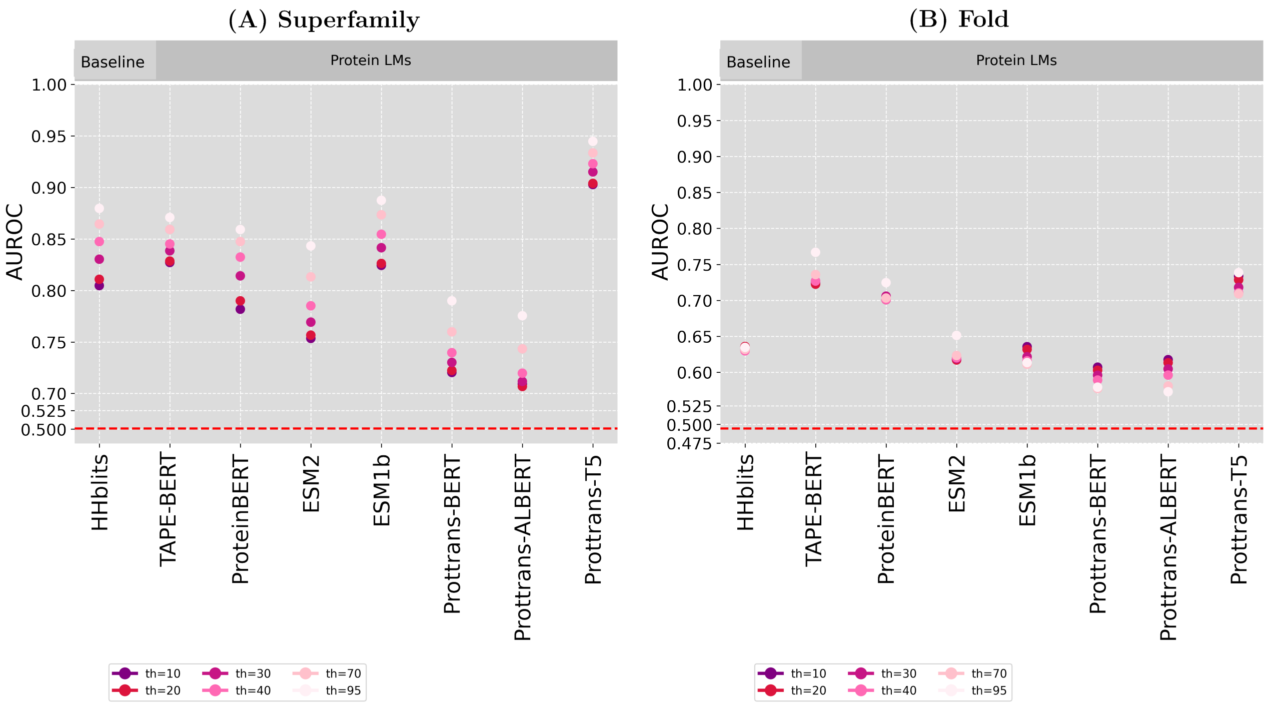

Fig. 3. Unweighted performance scores across Superfamily or Fold classes shows small deviations. Compare with the weighted scores shown in Figures 1 (A) and (B) .

different models across all sequence identity thresholds when the representative sequences are provided by the CD-HIT (Fu et al., 2012) clustering tool.

Detecting Fold-level remote homologs in SCOP2 dataset remains highly difficult. In terms of performance, no PLMs exceeded a Hit@10 score of 30% for datasets derived from SCOP2, with accuracy dropping to below 20% when computing Hit@1. When considering the datasets, it is notable that SCOPe encompasses fewer Folds per class (with 288, 173, 140, 393, and 98 Folds) compared to the SCOP2 classes (461, 240, 165, 519, and 104), nearly doubling for any specific class at a 95% sequence identity threshold. Moreover, minimal variance is observed in homology and remote homology detection performance based on SCOP2 datasets compared to those derived from SCOPe . Both the distribution of these datasets and the performance scores of the models suggest that SCOP2-derived Fold detection presents an even greater challenge for all protein language models across various sequence similarity thresholds, regardless of the underlying pre-training datasets and architectural biases of the models.

The PLMs that we considered still lag behind in detecting remote homologs at the Fold level when compared with their performance at the Superfamily level, as illustrated in Figure 1 (A) and (B) across various metrics. While three out of seven PLMs marginally surpass the 0.2 threshold in Hit@10, none of the models achieve such performance in Hit@1, indicating the persistent challenge in zero-shot detection of remote Fold homologs for future PLMs developed solely from sequence information.

In Figure 4 (C) and (D) we also show SCOP2 remote homology results when choosing representative sequences randomly instead of relying on the “representative” sequence recommended by CD-HIT. This can be compared with the results shown in Figure 4 (A) and (B) which relies on the CD-HIT provided representative sequence for each cluster.

Numerical Results Corresponding to Figure 1 (B)

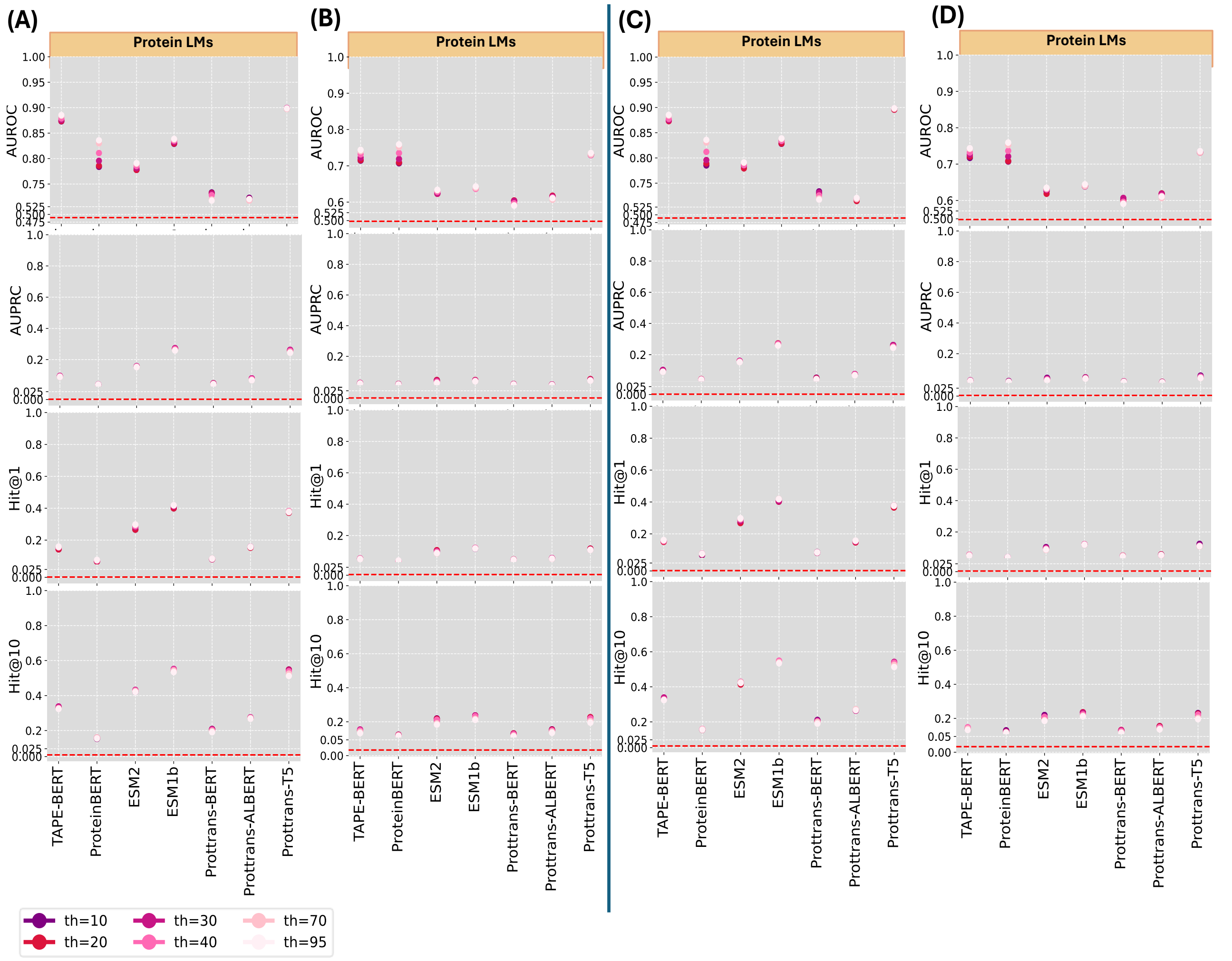

Fig. 4. SCOP2 performance comparison. (A) using the CD-HIT representative sequence for each cluster at different clustering identity thresholds. Superfamily-level remote homology detection scores are shown on the left and Fold-level scores are shown in (B). (C) Shows Superfamily-level scores when the using random representative sequence for each CD-HIT cluster at different clustering identity thresholds. (D) shows Fold-level results using random sequences from each cluster.

Threshold	HHblits	TAPE-BERT	ProteinBERT	ESM2	ESM1b	Prottrans-BERT	Prottrans-ALBERT	Prottrans-T5
**AUROC**								
@10%	0.83	0.86	0.79	0.79	0.85	0.75	0.72	**0.92**
@20%	0.84	0.86	0.80	0.80	0.86	0.76	0.73	**0.92**
@30%	0.85	0.88	0.83	0.81	0.88	0.77	0.74	**0.93**
@40%	0.86	0.89	0.86	0.84	0.90	0.79	0.77	**0.94**
@70%	0.88	0.91	0.89	0.87	0.92	0.82	0.80	**0.96**
@95%	0.88	0.92	0.90	0.88	0.93	0.83	0.82	**0.96**
**AUPRC**								
@10%	**0.42**	0.12	0.07	0.19	0.34	0.07	0.10	0.35
@20%	**0.45**	0.12	0.07	0.20	0.36	0.07	0.11	0.37
@30%	**0.51**	0.14	0.10	0.25	0.41	0.08	0.14	0.43
@40%	**0.58**	0.17	0.12	0.31	0.47	0.10	0.19	0.48
@70%	**0.66**	0.23	0.16	0.39	0.54	0.16	0.27	0.55
@95%	**0.70**	0.24	0.17	0.40	0.55	0.18	0.28	0.56
**Hit@1**								
@10%	0.48	0.16	0.10	0.31	**0.49**	0.10	0.18	0.47
@20%	0.51	0.17	0.11	0.33	**0.52**	0.11	0.20	0.50
@30%	**0.59**	0.21	0.15	0.42	0.58	0.14	0.26	0.57
@40%	**0.66**	0.26	0.19	0.51	0.64	0.20	0.34	0.64
@70%	**0.75**	0.38	0.27	0.64	0.74	0.33	0.50	0.74
@95%	**0.77**	0.41	0.30	0.66	0.76	0.37	0.54	0.76
**Hit@10**								
@10%	0.49	0.34	0.20	0.46	0.62	0.23	0.30	**0.64**
@20%	0.53	0.35	0.21	0.47	0.65	0.24	0.32	**0.66**
@30%	0.60	0.40	0.26	0.55	0.69	0.29	0.38	**0.71**
@40%	0.67	0.45	0.30	0.62	0.73	0.35	0.45	**0.75**
@70%	0.75	0.57	0.39	0.73	0.81	0.48	0.60	**0.82**
@95%	0.78	0.58	0.42	0.73	0.82	0.51	0.62	**0.83**

Table 5. Superfamily-level remote homology prediction results using SCOPe in tabular form. The same information is shown in Figure 1 (A).

Threshold	HHblits	TAPE-BERT	ProteinBERT	ESM2	ESM1b	Prottrans-BERT	Prottrans-ALBERT	Prottrans-T5
**AUROC**								
@10%	0.62	**0.75**	0.70	0.62	0.64	0.60	0.61	0.73
@20%	0.62	**0.75**	0.71	0.62	0.64	0.59	0.61	0.73
@30%	0.62	**0.76**	0.72	0.62	0.64	0.59	0.60	0.73
@40%	0.61	**0.76**	0.73	0.63	0.64	0.60	0.58	0.73
@70%	0.62	**0.78**	0.76	0.65	0.65	0.58	0.59	0.74
@95%	0.63	**0.79**	0.78	0.68	0.67	0.59	0.60	0.75
**AUPRC**								
@10%	**0.07**	0.04	0.03	0.03	0.06	0.02	0.02	**0.07**
@20%	**0.07**	0.04	0.03	0.03	0.06	0.02	0.02	**0.07**
@30%	**0.07**	0.05	0.03	0.03	0.06	0.02	0.02	0.06
@40%	**0.08**	0.05	0.02	0.04	0.06	0.02	0.02	0.07
@70%	**0.07**	0.05	0.02	0.03	0.06	0.02	0.02	0.06
@95%	**0.09**	0.04	0.02	0.03	0.06	0.02	0.02	0.06
**Hit@1**								
@10%	0.04	0.10	0.05	0.09	**0.15**	0.05	0.06	**0.15**
@20%	0.04	0.10	0.05	0.09	**0.15**	0.05	0.06	0.14
@30%	0.04	0.10	0.05	0.09	**0.15**	0.05	0.06	0.14
@40%	0.06	0.11	0.04	0.12	**0.17**	0.05	0.07	0.14
@70%	0.05	0.12	0.04	0.10	**0.16**	0.05	0.08	0.11
@95%	0.08	0.11	0.05	0.11	**0.16**	0.05	0.08	0.12
**Hit@10**								
@10%	0.06	0.25	0.13	0.20	**0.28**	0.14	0.16	0.27
@20%	0.06	0.24	0.13	0.20	**0.27**	0.14	0.16	**0.27**
@30%	0.06	0.25	0.14	0.20	**0.27**	0.13	0.16	0.26
@40%	0.07	0.24	0.15	0.22	**0.27**	0.13	0.16	0.23
@70%	0.07	0.24	0.15	0.22	**0.26**	0.12	0.17	0.22
@95%	0.15	0.24	0.13	0.21	**0.26**	0.11	0.17	0.21

Table 6. Fold-level remote homology prediction results using SCOPe in tabular form. The overall trend is illustrated in Figure 1 (B).
